# High-Throughput-Methyl-Reading (HTMR) assay: a solution based on nucleotide methyl-binding proteins enables large-scale screening for DNA/RNA methyltransferases and demethylases

**DOI:** 10.1093/nar/gkab989

**Published:** 2021-10-29

**Authors:** Senhao Xiao, Siqi Guo, Jie Han, Yanli Sun, Mingchen Wang, Yantao Chen, Xueyu Fang, Feng Yang, Yajuan Mu, Liang Zhang, Yiluan Ding, Naixia Zhang, Hualiang Jiang, Kaixian Chen, Kehao Zhao, Cheng Luo, Shijie Chen

**Affiliations:** The Center for Chemical Biology, Drug Discovery and Design Center, State Key Laboratory of Drug Research, Shanghai Institute of Materia Medica, Chinese Academy of Sciences, Shanghai 201203, China; School of Life Science and Technology, ShanghaiTech University, Shanghai 201210, China; University of Chinese Academy of Sciences, No.19A Yuquan Road, Beijing 100049, China; The Center for Chemical Biology, Drug Discovery and Design Center, State Key Laboratory of Drug Research, Shanghai Institute of Materia Medica, Chinese Academy of Sciences, Shanghai 201203, China; School of Pharmacy, Nanchang University, Nanchang 330006, Jiangxi, China; The Center for Chemical Biology, Drug Discovery and Design Center, State Key Laboratory of Drug Research, Shanghai Institute of Materia Medica, Chinese Academy of Sciences, Shanghai 201203, China; The Center for Chemical Biology, Drug Discovery and Design Center, State Key Laboratory of Drug Research, Shanghai Institute of Materia Medica, Chinese Academy of Sciences, Shanghai 201203, China; School of Pharmacy, Key Laboratory of Molecular Pharmacology and Drug Evaluation (Yantai University), Ministry of Education; Collaborative Innovation Center of Advanced Drug Delivery System and Biotech Drugs in Universities of Shandong, Yantai University, Yantai 264005, China; The Center for Chemical Biology, Drug Discovery and Design Center, State Key Laboratory of Drug Research, Shanghai Institute of Materia Medica, Chinese Academy of Sciences, Shanghai 201203, China; School of Life Science and Technology, ShanghaiTech University, Shanghai 201210, China; University of Chinese Academy of Sciences, No.19A Yuquan Road, Beijing 100049, China; The Center for Chemical Biology, Drug Discovery and Design Center, State Key Laboratory of Drug Research, Shanghai Institute of Materia Medica, Chinese Academy of Sciences, Shanghai 201203, China; The Center for Chemical Biology, Drug Discovery and Design Center, State Key Laboratory of Drug Research, Shanghai Institute of Materia Medica, Chinese Academy of Sciences, Shanghai 201203, China; School of Life Science and Technology, ShanghaiTech University, Shanghai 201210, China; University of Chinese Academy of Sciences, No.19A Yuquan Road, Beijing 100049, China; The Center for Chemical Biology, Drug Discovery and Design Center, State Key Laboratory of Drug Research, Shanghai Institute of Materia Medica, Chinese Academy of Sciences, Shanghai 201203, China; Department of Pharmacology and Chemical Biology, State Key Laboratory of Oncogenes and Related Genes, Shanghai Jiao Tong University School of Medicine, Shanghai 200025, China; Department of Pharmacology and Chemical Biology, State Key Laboratory of Oncogenes and Related Genes, Shanghai Jiao Tong University School of Medicine, Shanghai 200025, China; University of Chinese Academy of Sciences, No.19A Yuquan Road, Beijing 100049, China; Analytical Research Center for Organic and Biological Molecules, Shanghai Institute of Materia Medica, Chinese Academy of Sciences, Shanghai 201203, China; University of Chinese Academy of Sciences, No.19A Yuquan Road, Beijing 100049, China; Analytical Research Center for Organic and Biological Molecules, Shanghai Institute of Materia Medica, Chinese Academy of Sciences, Shanghai 201203, China; The Center for Chemical Biology, Drug Discovery and Design Center, State Key Laboratory of Drug Research, Shanghai Institute of Materia Medica, Chinese Academy of Sciences, Shanghai 201203, China; School of Life Science and Technology, ShanghaiTech University, Shanghai 201210, China; University of Chinese Academy of Sciences, No.19A Yuquan Road, Beijing 100049, China; The Center for Chemical Biology, Drug Discovery and Design Center, State Key Laboratory of Drug Research, Shanghai Institute of Materia Medica, Chinese Academy of Sciences, Shanghai 201203, China; School of Life Science and Technology, ShanghaiTech University, Shanghai 201210, China; University of Chinese Academy of Sciences, No.19A Yuquan Road, Beijing 100049, China; School of Pharmacy, Key Laboratory of Molecular Pharmacology and Drug Evaluation (Yantai University), Ministry of Education; Collaborative Innovation Center of Advanced Drug Delivery System and Biotech Drugs in Universities of Shandong, Yantai University, Yantai 264005, China; The Center for Chemical Biology, Drug Discovery and Design Center, State Key Laboratory of Drug Research, Shanghai Institute of Materia Medica, Chinese Academy of Sciences, Shanghai 201203, China; School of Life Science and Technology, ShanghaiTech University, Shanghai 201210, China; University of Chinese Academy of Sciences, No.19A Yuquan Road, Beijing 100049, China; School of Pharmaceutical Science and Technology, Hangzhou Institute for Advanced Study, UCAS, Hangzhou 310024, China; The Center for Chemical Biology, Drug Discovery and Design Center, State Key Laboratory of Drug Research, Shanghai Institute of Materia Medica, Chinese Academy of Sciences, Shanghai 201203, China; University of Chinese Academy of Sciences, No.19A Yuquan Road, Beijing 100049, China; School of Pharmaceutical Science and Technology, Hangzhou Institute for Advanced Study, UCAS, Hangzhou 310024, China

## Abstract

Epigenetic therapy has significant potential for cancer treatment. However, few small potent molecules have been identified against DNA or RNA modification regulatory proteins. Current approaches for activity detection of DNA/RNA methyltransferases and demethylases are time-consuming and labor-intensive, making it difficult to subject them to high-throughput screening. Here, we developed a fluorescence polarization-based ‘High-Throughput Methyl Reading’ (HTMR) assay to implement large-scale compound screening for DNA/RNA methyltransferases and demethylases-DNMTs, TETs, ALKBH5 and METTL3/METTL14. This assay is simple to perform in a mix-and-read manner by adding the methyl-binding proteins MBD1 or YTHDF1. The proteins can be used to distinguish FAM-labelled substrates or product oligonucleotides with different methylation statuses catalyzed by enzymes. Therefore, the extent of the enzymatic reactions can be coupled with the variation of FP binding signals. Furthermore, this assay can be effectively used to conduct a cofactor competition study. Based on the assay, we identified two natural products as candidate compounds for DNMT1 and ALKBH5. In summary, this study outlines a powerful homogeneous approach for high-throughput screening and evaluating enzymatic activity for DNA/RNA methyltransferases and demethylases that is cheap, easy, quick, and highly sensitive.

## Graphical Abstract

**Graphical Abstract:**
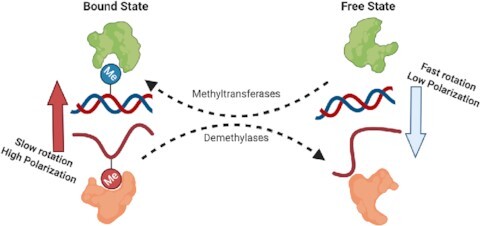
The principle of HTMR assay.

## INTRODUCTION

The modification of methylation on chromatin is an important epigenetic marker regulated by several kinds of methyltransferases and demethylases. DNA methylation, which primarily occurs at C-5 of cytosine of the CpG dinucleotide cluster, plays a vital role in several cell processes, including transcriptional regulation, genomic imprinting, and X-chromosome inactivation (1–4). In mammals, DNA methylation is regulated by DNA methyltransferases and demethylases. The DNA methyltransferase family proteins-DNMT1 and DNMT3A/DNMT3B are responsible for maintenance methylation and *de novo* methylation, respectively (5). In some cases, 5-methyl-cytosine (5mC) can be reversed to an unmodified state by the ten-eleven translocation family proteins-TET1, TET2 and TET3 (6). TETs catalyze the continuous oxidation of 5mC to 5hmC, 5fC and 5caC in the presence of Fe^2+^ and 2-oxoglutarate (2-OG), while oxidation products lead to the dilution of 5mC and can restore unmodified cytosine after DNA replication (7). Thymine DNA glycosylase (TDG) can also mediate DNA demethylation by the excision of 5fC and 5caC, along with base excision repair (BER) (8). Aberrant DNA methylation is involved in many human diseases and tumors at the developmental stage (9,10).

Methyl-modification can also occur in RNA, where N6-methyl-adenosine (m^6^A) on RNA has emerged as a new epigenetic modification with complicated biological functions (11). m^6^A is the first-known reversible and dynamic RNA methylation that participates in the regulation of RNA metabolism, including RNA processing, mRNA decay, and translation regulation (12–14). In mammals, m^6^A is primarily produced by a multicomponent methyltransferase complex containing METTL3, METTL14 and WTAP (15). FTO and ALKBH5, which belong to the α-ketoglutarate-dependent dioxygenase family, were identified as m^6^A demethylase and catalyze the demethylation of m^6^A in a 2-OG- and Fe^2+^-dependent manner (16,17). In addition, m^6^A and m^6^A-related proteins are involved in cellular differentiation, embryonic development, neurogenesis, and many other human diseases (13,14,18).

Epigenetic therapy is a promising strategy for cancer treatment. Many histone modification enzymes have already had potent and selective inhibitors with well-demonstrated binding mode and distinct mechanism of action, including EZH2 (19), Dot1L (20), p300 (21), PRMTs (22,23), SMYDs (24,25), LSD1 (26), KAT6A/B (27), KDM6B (28) and HDACs (29). The DNA/RNA methylation-related proteins have also proven to be potential drug targets for many diseases(10,30–32). However, few potent inhibitors have been reported, except FTO (33–35). Two cytidine analogs, Vidaza (5-azacytidine) and Decitabine (5-aza-2’-deoxycytidine) can robustly reverse DNA methylation and have been approved by the FDA to treat myelodysplastic syndrome (MDS) (36). These drugs have displayed significant promise for combination therapies (37–39), induced pluripotent stem cells (40,41), immunotherapy (42,43), and other biological uses. Vidaza and Decitabine can be incorporated into DNA and lead to covalent capture and degradation of DNMTs during S-phase replication *in vivo* (44). However, these cytidine analogs are not targeted inhibitors of DNMTs and can result in significant toxic side effects. TET family proteins are essential for the progression of many diseases due to a loss of 5hmC and DNA hypermethylation (32,45). Ascorbic acid, a natural TETs activator, also acts as an effective DNA demethylation agent by improving the activity of TETs and decreasing DNA 5mC levels *in vivo* (32,46,47). The proteins that modulate m^6^A are also promising drug targets and provide new insight for the treatment of various human diseases (13,14,18,30,31,48–51). Yang *et al.* have reported several potent FTO inhibitors, which have been used in hematological malignancy therapy (33–35,52). The discovery of small molecules that target m^6^A related proteins is a promising field of study with broad future applications.

The lack of a powerful high-throughput assay is one barrier that critically hinders the drug development of DNA/RNA methyltransferases and demethylases. It is hard to detect and quantify the methyl group in DNA/RNA directly using traditional methods such as UV/vis absorbance and fluorescence intensity. Some SAH-coupled kinetic assays (53,54) have low sensitivity and require high concentrations of substrates and enzymes. While radioisotopes are highly sensitive and are the gold-standard assays for methylation reactions (55), this method is inadequate for demethylation reactions. The demethylation activity of TET2 or ALKBH5/FTO was evaluated by HPLC analysis, where a single sample was allowed to be detected at a time (34,56). ELISA is a classic antibody-based method that is non-homogeneous and low-throughput. The current assays mentioned above require at least one washing or enzyme digestion step that cannot be directly applied to automated and large-scale screenings due to the high cost and difficulty of operation. Therefore, the design and development of a suitable high-throughput assay can help accelerate the development of DNA/RNA methyltransferases and demethylases drugs.

**Figure 1.**
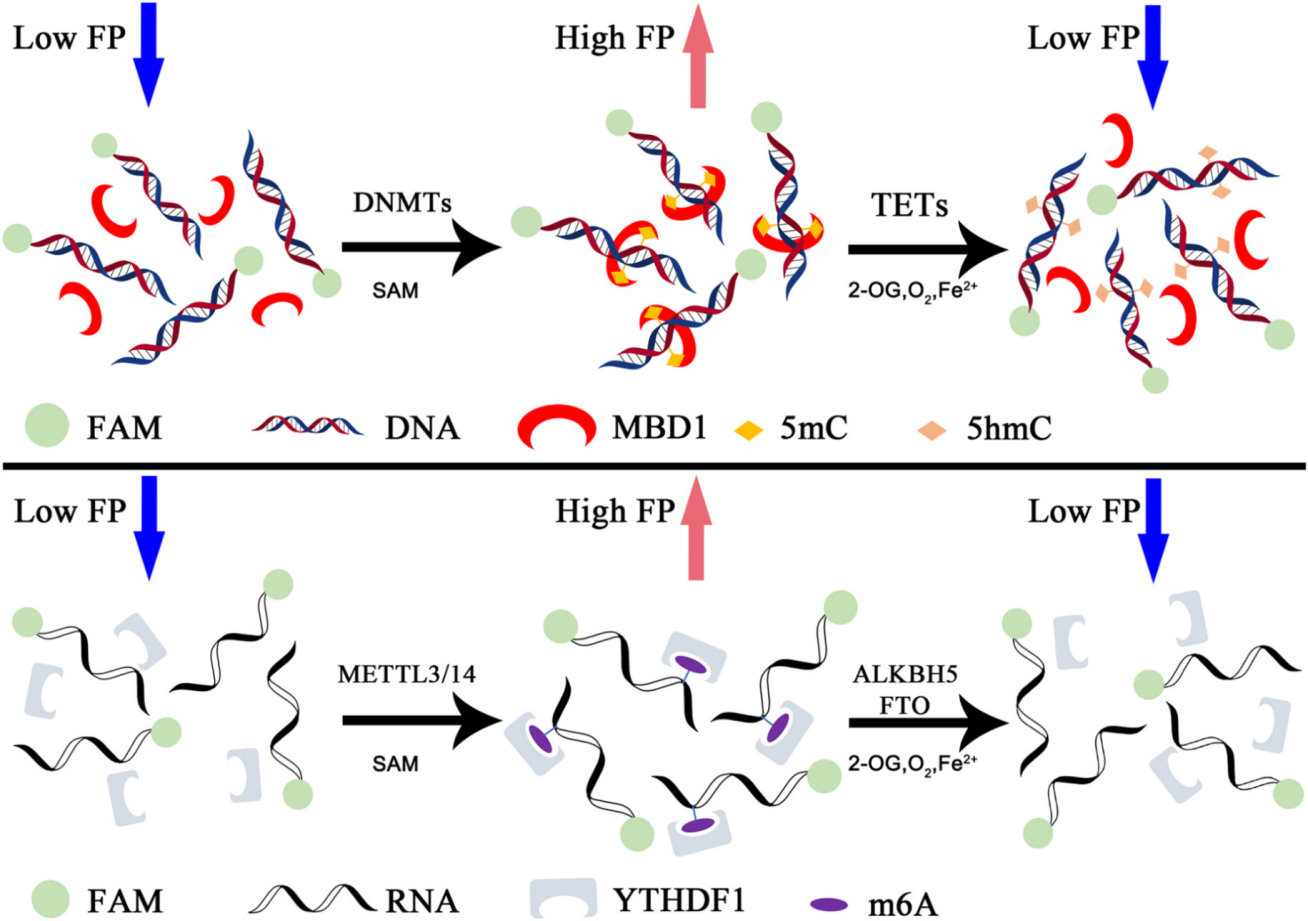
Diagram of assay working principle. FAM-labeled oligonucleotide produced different FP signals at different methylation statuses. Methylation can be converted by enzymes in the presence of cofactors.

In this study, we developed an FP-based homogeneous assay for DNA/RNA methyltransferases and demethylases, which can be applied to DNMTs, TETs, METTL3/14 and ALKBH5/FTO. The assay was easy to perform under both automatic and manual operation with a single step, adding the methyl-binding proteins MBD1 or YTHDF1. The different methyl statuses of fluorescence-labeled oligonucleotides can be distinguished by the FP signal produced by methyl-binding proteins. As the methyl status changes, the extent of the reaction can be recorded by a variation in FP signals (Figure 1). This assay is robust, has a high *Z*′ factor, and has been successfully used to identify two natural product inhibitors of DNMT1 and ALKBH5 through high-throughput screening. Furthermore, it is feasible to perform the cofactor competition assay to investigate the mechanism of action of candidate compounds. Overall, this paper provides an approach to identify small–molecular inhibitors of DNA/RNA methyltransferases and demethylases, and evaluate their enzymatic inhibitory activity.

## MATERIALS AND METHODS

### Preparation of oligonucleotides

All oligonucleotides were chemically synthesized by GeneScript Biotech. The oligonucleotides were dissolved in nuclease-free water (Nalgene) at a concentration of 100 μM. DNA single-stranded primers were annealed at 95°C for 7.5 min, then inserted into a foam box and cooled to room temperature.

The 12-bp DNA oligonucleotides sequences were as follows: 5′-FAM-TAXGACCAGGAT-3′ (top strand) and 5′-ATCCTGGTXGTA-3′ (bottom strand), where X represents C, 5mC, 5hmC or 5caC. The 16-nt RNA oligonucleotides sequence was 5′-FAM-GAACCGGYCUGUCUUA-3′, where Y represents A or m^6^A (57). The oligonucleotides used in this study are summarized in Supplementary Table S1.

### Protein expression and purification

All constructs (except human DNMT1) were expressed in an *E. coli* BL21(DE3) codon plus RIPL strain. The full-length METTL3 and METTL14 plasmids were obtained from Professor Ping Yin, College of Life Science and Technology, HuaZhong Agricultural University, Wuhan. The untagged MBD1 (1–105) and ALKBH5 (66–292) catalytic domain genes were subcloned into pET28b and pET28a vectors, respectively. The human TET2 catalytic domain (1129–1936, 1481–1843 was replaced by GGGGSGGGGSGGGGS) gene was subcloned into pET28b vector. Genes encoding tri-MBD1 (tandem three MBD1 1–105 ORF), YTHDF1 (361–559), YTHDF2 (380–579) and YTHDC1 (355–492) were chemically synthesized by Generay Biotech and subcloned into the pGEX-6P-1 vector. The human DNMT1 (351–1600) gene with N terminal 6xHis tag and a TEV cleavage site was subcloned into a modified pFBDM vector and expressed in *Spodoptera frugiperda*. The untagged MBD1 (58) (the same as Tri-MBD1), METTL3/14 (59,60), ALKBH5 (61) and TET2 (56,62) were expressed and purified as previously described.

For YTHDF1, YTHDF2 and YTHDC1, the cells were cultured in an LB culture at 37°C until OD600 reached 0.8. The cells were then induced by 0.4 mM IPTG for 16 hours after the temperature reached 16°C. The protein was initially purified using GSTrap (GE healthcare) affinity chromatography, followed by ion-exchange chromatography and gel filtration chromatography in a buffer containing 20 mM HEPES, pH 7.4, 250 mM NaCl, 5% glycerol and 2 mM DTT. For DNMT1 (351–1600), the recombinant baculovirus were obtained according to BAC-TO-BAC^®^ Baculovirus Expression System (Invitrogen) standard procedures. Sf9 insect cells were infected with a high-titer recombinant baculovirus stock for 72h to overexpress the recombinant proteins. The cells were then harvested and resuspended in a lysis buffer (50 mM Tris–HCl, pH 8.0, 500 mM NaCl, 5 mM β-mercaptoethanol and 1 mM PMSF) and lysed using a high-pressure cell disruptor. Insoluble material was removed by centrifugation at 18000rpm. The supernatant was loaded onto a HisTrap column (GE Healthcare) and washed with wash buffer (50 mM Tris–HCl, pH 8.0, 500 mM NaCl, 5mM β-mercaptoethanol and 20 mM imidazole). The protein was then eluted with an elution buffer (20 mM Tris–HCl, pH 8.0, 500 mM NaCl, 5 mM β-mercaptoethanol, and 250 mM imidazole). The proteins were further purified by ion-exchange chromatography and gel filtration chromatography in an SEC buffer containing 20 mM HEPES, pH 7.4, 100 mM NaCl, 1 mM DTT. The proteins were concentrated, flash-frozen in liquid nitrogen, and stored at −80°C. The yield of human DNMT1 was ∼12 mg per liter. All procedures were performed at 4°C or on ice.

### Z’ factor determination

Z’ factor was determined to assess the performance of DNMT1, TET2, METTL3/14 and ALKBH5 assays for high-throughput screening (63). Cofactor concentrations were as follows: 1 μM SAM (DNMT1), 1 μM SAM (METTL3/14), 1 mM 2-OG (TET2) and 200 μM 2-OG (ALKBH5). The assays were performed with 50 positive controls and 50 negative controls (array in 5 columns and 10 rows) in 384 well formats. The *Z’* factor was calculated by the following formula:

$$\begin{equation*}{\rm{Z^{\prime}}} = \ 1 - \frac{{3{{\rm{\sigma }}_{\rm{P}}} + 3{{\rm{\sigma }}_{\rm{N}}}}}{{\left| {{{\rm{\mu }}_{\rm{P}}} - {{\rm{\mu }}_{\rm{N}}}} \right|}}\end{equation*}$$

- σ_P_ represents the standard deviation of the positive group;
- μ_P_ represents the mean of the positive group;
- σ_N_ represents the standard deviation of the negative group;
- μ_N_ represents the mean of the negative group.

### Time-coursed assay

The time-coursed assay was performed to plot the time-signal curve. The 10 μl mixer of oligonucleotide and cofactors was transferred into wells. The reactions were initiated by adding different concentration enzymes at different time points: 0, 10, 20, 30, 40 and 50 min, resulting in respective reaction times of 60, 50, 40, 30, 20 and 10 min. Methyl-binding proteins were then added into the wells to terminate the reaction and standardize the incubation time. Wells with a reaction time of 0 min were slightly different: we added methyl-binding proteins to prevent the conversion and then added the enzymes. Cofactor concentrations were as follows: 1 μM SAM (DNMT1), 2.5 μM SAM (METTL3/14), 1 mM 2-OG (TET2) and 200 μM 2-OG (ALKBH5). All samples were performed in triplicate. The signals were read on EnVision (PerkinElmer). And curves were plotted in the Graphpad prism 7.0.

### Assay development and optimization

To start the assay, the binding curve of the substrate oligonucleotide and the product oligonucleotide (chemical synthetic) were titrated to determine the proper concentration of methyl-binding proteins. The final NaCl conditions of DNMT1, TET2, METTL3/14 and ALKBH5 were 400, 300, 250 and 250 mM, respectively. The assays could be performed in 10 μl + 10 μl, 20 μl + 20 μl (as in this study), or other formats in a black 384-well plate (Corning #3575). The former is the reaction volume containing enzyme, oligonucleotide, and cofactors, while the latter is the volume of methyl-binding protein. The reaction was initiated by the addition of the enzymes or the substrates and terminated by the addition of methyl-binding proteins. The FP signals were then measured in fluorescence polarization standard mode by EnVision Multilabel Reader (PerkinElmer) after 10–15 min of incubation. The plate can be repeatedly measured. Total fluorescence intensity (FI) can be automatically calculated by the EnVision FP module and is expected to be approximately the same, though results demonstrating FI changes of 30% should be critically analyzed, as this could be the result of improper pipetting or the interference of dose-dependent compounds. Additionally, this experiment must avoid strong light exposure. All data were analyzed in GraphPad Prism 7.0.

False-positive compounds should be abandoned during the high-throughput screening. Compounds that cause aberrant FP values or fluorescence quenching with significantly decreased total FI values are most likely DNA/RNA binders. The quenching caused by these compounds can be eliminated by the use of a high concentration of unlabeled oligonucleotide (Supplementary Figure S7), while some compounds can influence the binding assay of methyl-binding proteins. For example, a high concentration (over 50 μM) of IOX1 can inhibit the binding of tri-MBD1, but not YTHDF1. In these cases, hit compounds in the primary screen must be confirmed by the following test to exclude false-positive compounds. This entails replacing half of the substrate oligonucleotide with product oligonucleotide in the absence of the enzyme and adding the methyl-binding protein into the wells. The FP signal can then be measured according to the above steps. In these cases, false-positive compounds that demonstrate activity during the screening can still influence the FP signal value, while true hits will not.

#### DNMT1

The reaction components (enzyme, oligonucleotide and cofactors) were diluted in a reaction buffer containing 20 mM HEPES, pH 7.4, 1 mM EDTA and 1 mM DTT. For IC_50_ determination, 5 μl SAH was pre-incubated with 20 nM DNMT1(351–1600) for 15 min, after which a 10 μl mixer of 20 nM FAM-labeled hemi-methylated DNA and different concentrations of SAM were added to initiate the reaction. The plate was incubated at 37°C for 70 min, after which 20 μl tri-MBD1(1.5 μM as final concentration) protein (which was diluted in 20 mM HEPES, pH7.4, 800 mM NaCl) was transferred to the wells to end the reaction. This was followed by incubation for 10–15 min at RT (room temperature), after which the plate was read by EnVision (PerkinElmer).

#### METTL3/METTL14

The enzymes, oligonucleotides, and cofactors were diluted in a reaction buffer containing 20 mM HEPES, pH 7.4, 1 mM DTT and 0.4 U/μl RNase inhibitor (BBI Life sciences). For IC_50_ determination, 5 μl SAH was pre-incubated with 40 nM METTL3/14 for 15 min. A 10 μl mixer of 40 nM FAM-labeled unmodified GGACU motif-containing RNA and different concentrations of SAM were added to initiate the reaction. The plate was then incubated at RT for 60 min. We then transferred 20 μl GST-YTHDF1(750 nM as final concentration) protein, which was diluted in 20 mM HEPES, pH 7.4, 500 mM NaCl, to the wells to end the reaction. After 10–15 min of incubation, the plate was read by Envision (PerkinElmer).

#### TET2

The reaction buffer containing 20 mM HEPES, pH 8.0, 100 mM NaCl, 1 mM DTT, 1 mM ATP, 1 mM 2-OG, 2 mM ascorbic acid and 100 μM Fe_2_(NH_4_)_2_(SO_4_)_2_. Fe^2+^ is the last to be added. The reaction contained 40 nM TET2 and 20 nM FAM-labeled fully-methylated DNA occurred at 37°C for 60 min. Next, 20 μl tri-MBD1 (500 nM as final concentration) protein was diluted in 20 mM HEPES, pH 7.4, 500 mM NaCl and transferred into wells to end the reaction. After 10–15 min of incubation, the plate was read by Envision (PerkinElmer).

#### ALKBH5

The reaction buffer containing 20 mM HEPES, pH 7.4, 250 mM NaCl, 200 μM 2-OG, 300 μM ascorbic acid, and 50 μM Fe_2_(NH_4_)_2_(SO_4_)_2_. Fe^2+^ is the last to be added. 40 nM FAM-labeled m^6^A-modified GGACU motif-containing RNA and 20 nM ALKBH5 was used in the reaction. The reaction was performed at RT for 50 min. 20 μl GST-YTHDF1 (750 nM as final concentration) was diluted in a buffer containing 20 mM HEPES, pH 7.4, 250 mM NaCl and added to the wells to end the reaction. The plate can then be read immediately or after 10–15 min of incubation due to an equal concentration of NaCl. After this, the plate can be read by EnVision (PerkinElmer).

All the curves were plotted in the Graphpad prism 7.0. And the IC_50_ values were determined by the log(inhibitor) versus response—variable slope (four parameters) model in Graphpad prism 7.0. The Ki determined by the intersection of the straight-line and y-axis in the IC_50_–conc. substrate curve, derived by Cheng-Pursoff equation (64):

$$\begin{equation*}{{\rm IC}_{50}} = {K_{\rm i}}\ \cdot \left( {1 + \frac{{\left[ {\rm S} \right]}}{{{K_{\rm m}}}}} \right)\end{equation*}$$

- *K*_i_ is the inhibition constant of substrate competitive inhibitor;
- [S] represents the concentration of the substrate;
- *K*_m_ represents the Michaelis constant of the substrate;

### Enzymes FP binding assay

The FP binding assay of DNMT1 and METTL3/14 was performed in a buffer containing 20 mM HEPES, pH 7.4, and 1 mM DTT. The FP binding assay of TET2 and ALKBH5 was performed in the reaction buffer that removes the NaCl. At high concentrations of NaCl (over 100 mM), weak FP signals were detected for the enzymes.

### NMR spectroscopy

Saturation transfer difference (STD) and Carr-Purcell-Meiboom-Gill (CPMG) experiments were performed to investigate the interaction between the compound and the protein, as previously described (65). All NMR spectra were acquired on a Bruker Avance III 600 MHz NMR spectrometer equipped with a cryoprobe (Bruker Biospin, Germany). The 5 μM ALKBH5(66–292) protein and 200 μM compound were dissolved into a phosphate buffer containing 20 mM NaH_2_PO_4_, 20 mM Na_2_HPO_4_, pH 7.4, 100 mM NaCl, 95% D_2_O, 5% DMSO-*d*_6_ and then used in NMR data acquisition. Transverse relaxation-edited spectra were recorded using the water-suppressed CPMG pulse sequence. Sixty-four transients were collected into 48 076 data points with a spectral width of 8.0 kHz and an acquisition time of 3.0 s. STD spectrum was recorded using the pulse sequence of stddiffgp19.3 with water suppression (3–9–19 WATERGATE). The on and off resonance excitation positions were defined at –0.17 and 33 ppm, respectively. The bandwidth for the saturation pulse was set to 42.4 Hz. One hundred and twenty-eight transients were collected into 32,768 data points with a spectral width of 9.6 kHz and an acquisition time of 1.71 s.

### Thermal shift assay

The thermal shift assay was performed with a QuantStudio 6 Flex real-time PCR machine (Applied Biosystems) with a buffer containing 20 mM HEPES, pH 7.4 and 100 mM NaCl. The final system was 20 μl, which contained 5 μM ALKBH5(66–292) protein, 1:1000 dilution of SYPRO Orange dye (purchased from Invitrogen, 5000× stock in DMSO), and DMSO or 100 μM compounds (final DMSO concentration was 0.1%). The samples were heated from 25 to 95°C (2.5°C/min). All samples were performed in triplicate and data were analyzed by Protein Thermal Shift Software version 1.3 (Applied Biosystems).

### Kinetic parameters determination

During the kinetic parameters determination experiments, the DNMT1 methylation reaction was performed in 20 mM HEPES, pH 7.4, 1 mM DTT, 1 mM EDTA at 37°C. The TET2 demethylation reaction was performed in 50 mM HEPES, pH 8.0, 100 mM NaCl, 100 μM Fe^2+^(NH4)_2_(SO4)_2_, 2 mM ascorbate acid, 1 mM DTT and 1 mM ATP at 37°C. The METTL3/14 methylation reaction was performed in 20 mM HEPES, pH 7.4, 1 mM DTT, 1 mM EDTA at 37°C. The ALKBH5 demethylation reaction was performed in 20 mM HEPES, pH 7.4, 300 μM ascorbate acid, 100 μM Fe^2+^(NH4)_2_(SO4)_2_ at 37°C. The signals were read and collected on EnVision Multilabel Plate Reader (PerkinElmer). And curves were plotted in the Graphpad prism 7.0.

### HPLC quantitative experiment

During the HPLC quantitative experiment, the reaction buffer of DNMT1 and METTL3/14 excluded EDTA to avoid the inhibition of Nuclease P1 and Alkaline Phosphatase. Four samples contained 4 μM fluorescence-labeled oligonucleotides, which reacted at different stages, along with the negative control samples (no SAM control for methyltransferase and no 2-OG control for demethylases, which represents 0% reaction) and positive control (equal amounts of corresponding products DNA/RNA instead of substrate DNA/RNA, which represents 100% reaction). A small part of the sample was removed to be detected by our assay, and the remainder was immediately terminated at 95°C. After 10 min of denaturation, the samples were digested by 100 U Nuclease P1 (NEB, #M0660S, 37°C for 3 h) and 2 U alkaline phosphatase (NEB, #M0371S, 37°C for 12 h). The digest products were then subjected to HPLC system with a Phenomenex Luna 5μ C18 analyses column (150 × 4.6 mm). Standard samples of N6-methyladenosine (Med Chem Express, #HY-N0086) and 5-methyl-2′-deoxycytidine (Med Chem Express, #HY-W012078) were used to plot standard curves to quantify the variation of m^6^A and 5mC, respectively. The mobile phase was A: 25 mM NaH_2_PO_4_, B: acetonitrile, with linear gradient elution from 5% to 95% at flow a rate of 1mL/min. The substrate conversion was quantified using the following formula:

$$\begin{equation*}{\rm Substrate}{\rm{\ }}{\rm conversion}{\rm{\% \ }} = \frac{{{\rm S} - {{\rm S}_{\rm N}}}}{{{{\rm S}_{\rm P}} - {{\rm S}_{\rm N}}}}{\rm{\ }} \times 100{\rm{\% }}\end{equation*}$$

- S represents the signal value in HPLC or our assays;
- S_N_ represents the signal value of negative control in HPLC or our assays;
- S_P_ represents the signal value of positive control in HPLC or our assays.

## RESULTS

### Detection of methylation reaction through coupling with an FP binding assay

Determining DNA methyltransferase and demethylase activity using typical radioisotope, ELISA, and HPLC methods is time-consuming, expensive and incapable of screening large-scale compounds. UHRF1 is a chromatin-binding protein that regulates DNA methylation and strongly binds to hemi-methylated DNA, rather than un-methylated DNA and fully-methylated DNA (66). The binding preference of UHRF1 suggests that UHRF1 could be an indicator that specifically recognizes hemi-methylated DNA, the substrate of DNMT1. Fluorescence polarization (FP) is a homogenous technology widely used to monitor biomolecular interactions (67,68). Some of the associations formed from the fluorescence-labeled hemi-methylated DNA with UHRF1 can be determined using the FP signal value (67). This suggests that the methylation reaction can be coupled with the binding state of UHRF1 and determined by variations in the FP signal. The signal is based on the variation of hemi-methylated DNA, but not on the cofactor SAM. DNA demethylation catalyzed by TETs can be analyzed similarly.

To explore the feasibility of this process, we calculated the theoretical FP signal value with 0% (only substrate) and 100% (only product) substrate conversion. The ΔFP value is the difference of the affinity curve of the methyl-binding protein and the substrate or product oligonucleotide and is considered as the signal window. mP_max_ and mP_min_ represent the maximum and minimum FP signal in the FP binding assay of the methyl-binding protein, respectively. *K*_A_ and *K*_B_ are the dissociation constants of the substrate and product, respectively (assuming the protein prefers product, *K*_A_ > *K*_B_). Enzyme binding was ignored in the calculation. The protein demonstrates a different affinity for the substrate and product and results in the discrepancy of the FP signal, as long as *K*_A_≠*K*_B_. The maximum ΔFP signal appears while the concentration of methyl-binding protein $[ {\rm{P}} ] = \sqrt {{{{K}}_{\rm{A}}} \cdot {{{K}}_{\rm{B}}}}$ (Supplementary Figure S3a). The protein will still bind to the substrate and produce the background FP signal. The non-saturated binding of the product would make partly dissociation of the association form. *K*_A_/*K*_B_ is an important parameter and is expected to be a large value. Assuming *K*_A_>> *K*_B_, the protein can act as an antibody in a wide range of concentrations that had no detectable binding with the substrate, and saturated binding with the product. Under these circumstances, the relationship between the FP signal and the extent of the reaction was considered linear (Supplementary Figure S3b), meaning that the FP signal can be used to directly calculate the conversion of the substrate.

### Methyl-binding proteins determination in assay development

The theoretical calculation demonstrated the feasibility of the assay, which was followed by a series of experiments to characterize the methyl-binding proteins. Previous study demonstrated that the preference of UHRF1 for fully-methylated DNA and hemi-methylated DNA is ∼10-fold (58). Low selectivity makes for a low signal window and high background. Larger *K*_A_/*K*_B_ and mP_max_–mP_min_ of the methyl-binding protein are required for the best assay performance. Methyl-CpG-binding domain (MBD) family proteins were identified as methyl-DNA binding proteins and found to possess a high preference for methylated DNA, especially fully-methylated DNA. And the main product of TETs demethylation reaction is fully-hydroxymethyl DNA, which binds weakly with MBD1 (58). We then purified the recombinant MBD1 (1–105) protein in the assay. The preference of MBD1 for fully-methylated DNA was insufficient (Supplementary Figure S1A), while the MBD1 protein was too small (only 11KDa) and produced a low mP_max_–mP_min_ during its FP binding assay.

**Figure 2.**
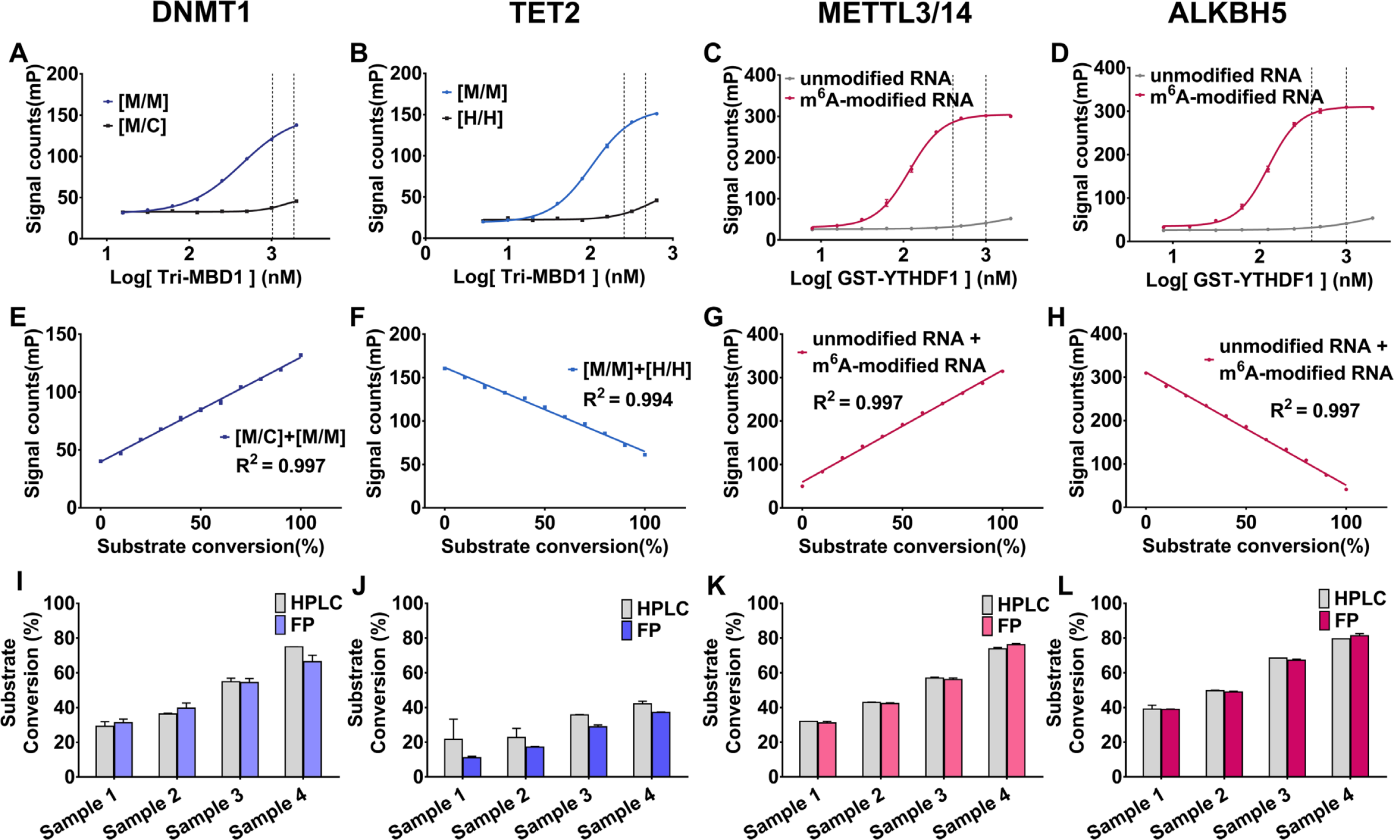
Titration (**A**–**D**) and standard curves (**E**–**H**) of the methyl-binding proteins to substrate/product oligonucleotides in the presence of 20 nM enzymes for DNMT1, TET2, METTL3/14 and ALKBH5. (A, E) Hemi-methylated DNA [M/C] versus fully-methylated DNA [M/M], (B, F) fully-methylated DNA [M/M] versus fully-hydroxymethylated DNA [H/H], (C, G) unmodified RNA versus m^6^A-modified RNA, (D, H) m^6^A-modified RNA versus unmodified RNA. M, H, C represent methylated, hydroxymethylated, and unmodified cytosine in dsDNA, respectively. Proper concentrations of methyl-binding proteins were between the two dotted lines and used in the plotting of standard curves. Parallel methylation/demethylation assays (HPLC quantification) of (I) DNMT1, (J)TET2, (K)METTL3/14, (L)ALKBH5. compared to this study were measured. Samples 1, 2, 3, 4 were obtained at different reaction stages. Error bars are displayed as mean ± SD. HPLC experiments were performed in duplicate and other experiments were performed in triplicate.

To improve the signal, we designed an optimized protein with three tandem MBD1 (1–105) ORFs and produced a recombinant GST-MBD1-MBD1-MBD1 protein (denoted as tri-MBD1). The protein has a higher mP_max_–mP_min_ (∼100 mP), while the preference of tri-MBD1 for fully-methylated DNA is poor in 150 mM NaCl but higher in higher ionic strength (Figure 2A, B, Supplementary Figure S1B). Under these conditions, tri-MBD1 can act as an antibody of fully-methylated DNA during the 5mC methylation or demethylation reaction (Figure 1). Next, we plotted the standard curve of the FP signal value versus the gradient proportion 0–100% mixer of fully-methylated DNA with hemi-methylated or fully-hydroxymethylated DNA, which represents the relative amount of substrate converted. This demonstrates that the curves of the different fraction mixtures of fully-methylated and hemi-methylated DNA yielded a linear correlation coefficient (*R*^2^) of 0.997 (Figure 2E), while the *R*^2^ of various fraction mixtures of fully-methylated and fully-hydroxymethylated DNA is 0.994 (Figure 2F). Both figures are consistent with our theoretical calculation, demonstrating that the FP signal can directly indicate the extent of the reaction.

Similarly, the same principle of the development of DNA methylation-related enzyme assay can be applied to RNA m^6^A methyltransferase METTL3/14 and demethylase ALKBH5 and FTO, the substrates and products of which can be distinguished by m^6^A binding proteins-YTH family proteins (69) (Figure 1). As such, we purified the YTH domain of YTHDF1, YTHDF2, YTHDC1, and plotted their binding affinity to m^6^A-modified RNA and un-methylated RNA. Over 100-fold selectivity makes YTHDF1 an antibody of m^6^A-modified RNA in *in vitro* assay (Figure 2C, D, Supplementary Figure S1D). Furthermore, when GST-YTHDF1 contained the N-terminal GST tag, it retained the same *K*_A_/*K*_B_ and had a much larger mP_max_–mP_min_ (∼250 mP). A proper concentration of GST-YTHDF1 was selected to plot the standard curve, which was linear with *R*^2^ = 0.997 (Figure 2G, H).

Altogether, the optimized tri-MBD1 and GST-YTHDF1 proteins have high selectivity between the substrates and products and generate a large signal window for detection. Our experimental results are consistent with theoretical calculations, demonstrating the efficacy of the assay.

### Ionic strength is essential for the assay

As the substrate, the fluorescent-labeled oligonucleotides can form stable complexes with the enzymes, while the enzyme-binding forms produce meaningless FP signals, which cause interference and must be eliminated. We plotted the binding affinity of the enzymes to their respective substrates and products and found that the methyltransferase DNMT1 and METTL3/14 demonstrated strong binding in their reaction buffer that contained no NaCl. However, virtually no binding was detected in a buffer containing 150 mM or higher concentrations of NaCl (Supplementary Figure S2A–F). Previous studies have demonstrated that NaCl could strongly inhibit the activity of mouse DNMT family proteins and METTL3/14 (70–73). DNMT1 and METTL3/14 can both tolerate high ionic strength *in vivo*. For the demethylases-TET2 and ALKBH5, the enzyme binding FP signals were also very weak in the high-NaCl buffer (Supplementary Figure S2G, H). To our knowledge, ionic strength is also important for the binding of the methyl-binding protein. MBD1 and YTHDF1 demonstrate little preference for methylated DNA/RNA at low concentrations of NaCl. Their preferences are exhibited at high ionic strengths of 150 mM NaCl or above (Supplementary Figure S1A, E). Therefore, the proper ionic strength must be titrated to achieve high selectivity. The modified protein tri-MBD1 demonstrates no preference for methyl-modified DNA at 150 mM NaCl and a high preference for methyl-modified DNA over 300 mM NaCl (Figure 2A, B, Supplementary Figure S1B). YTHDF1 demonstrates complete selectivity at 250 mM NaCl (Figure 2C, D). To distinguish between substrate and product, a proper high concentration of NaCl is required for the final detection of methyl-binding proteins to be highly selective. Meanwhile, high ionic strength eliminates the background FP signals produced by the binding of the enzymes (Supplementary Figure S2). These reactions could be terminated by high concentrations of NaCl (DNMT1, METTL3/14) or saturated binding of the substrate (TET2, ALKBH5) by adding methyl-binding protein to a stop buffer containing high ionic strength.

### Assay performed in DNMT1, TET2, METTL3/14 and ALKBH5

**Figure 3.**
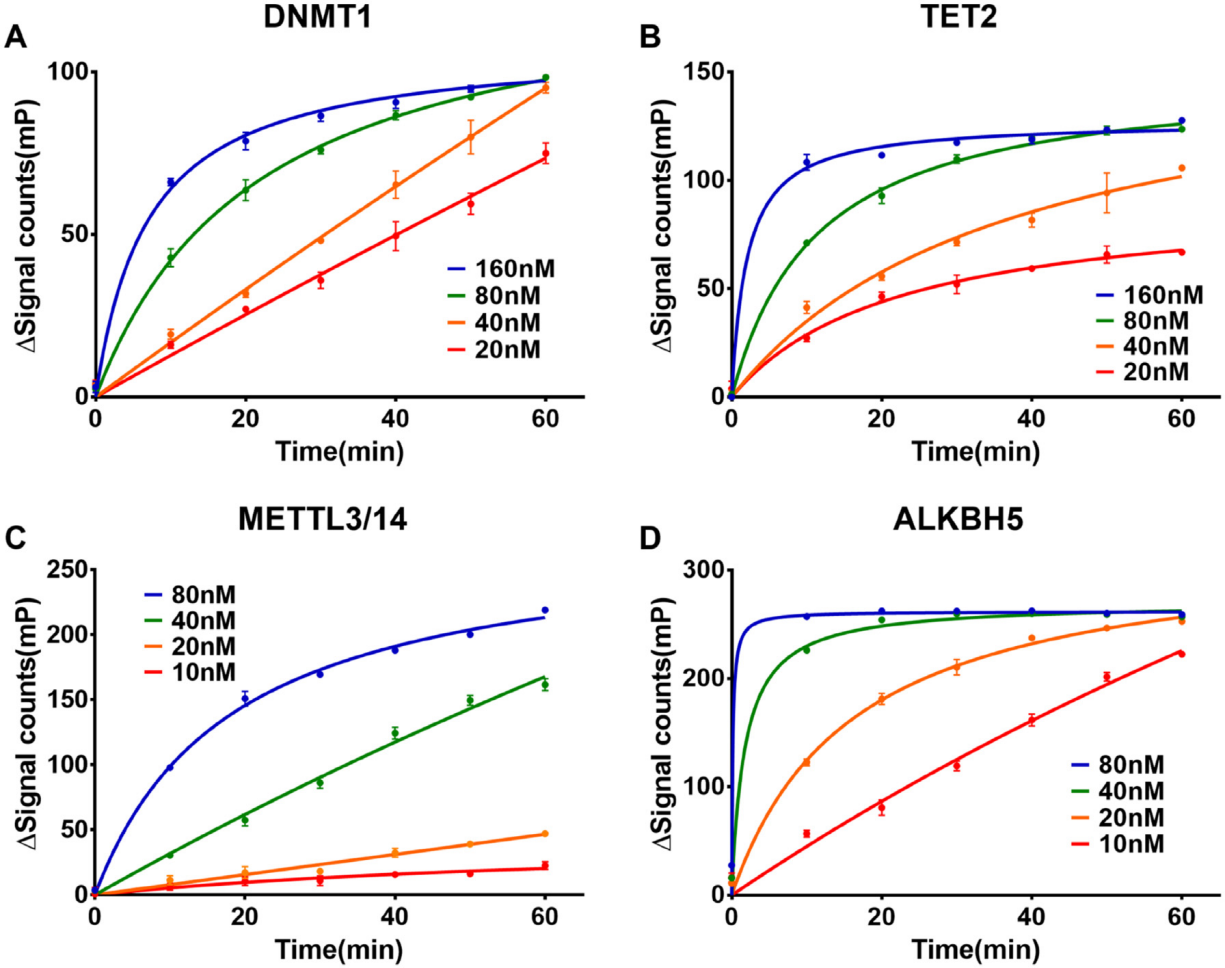
Time-course signal curve of (**A**) DNMT1, (**B**) TET2, (**C**) METTL3/14 and (**D**) ALKBH5. Reaction times were set to 0, 10, 20, 30, 40, 50 and 60 min. Proteins were diluted at four concentrations. All samples were performed in triplicate.

After determining the proper ionic strength and the optimal methyl-binding protein, we performed the enzyme reaction of DNMT1, TET2, METTL3/14 and ALKBH5 with our assay. Enzyme titration and time-course assay were performed to determine the reaction time and concentration of the enzymes (Figure 3). The variation of the FP signal increased as the reactions progressed and sharply increased as protein concentrations increased, confirming the assays were working. To ensure the FP signal accurately reflected the conversion of the substrate, we performed a quantitative high-performance liquid chromatography (HPLC) assay along with our method. As shown in Figure 2I–L, the results obtained from our assay were similar to those obtained from HPLC. Next, we performed the kinetic assay to confirm the ability of our assay to accurately replicate the kinetic parameters of the enzymes reported in other studies. As expected, the kinetic parameters determined from our assay are close to those identified by previous studies (Supplementary Figure S6) (62,71,74), although the *k*_cat_ for DNMT1 & METTL3/14 show difference which is possibly a result of different construct and origin of the enzymes. However, the reaction rates of METTL3/14 and ALKBH5 first increased and then decreased in the RNA *K*_m_ assays. These results were abnormal, although the *V*_0_ increased linearity at low concentrations of RNA. This could be because the single-strand RNA formed a dimer that could not be further methylated/demethylated.

**Figure 4.**
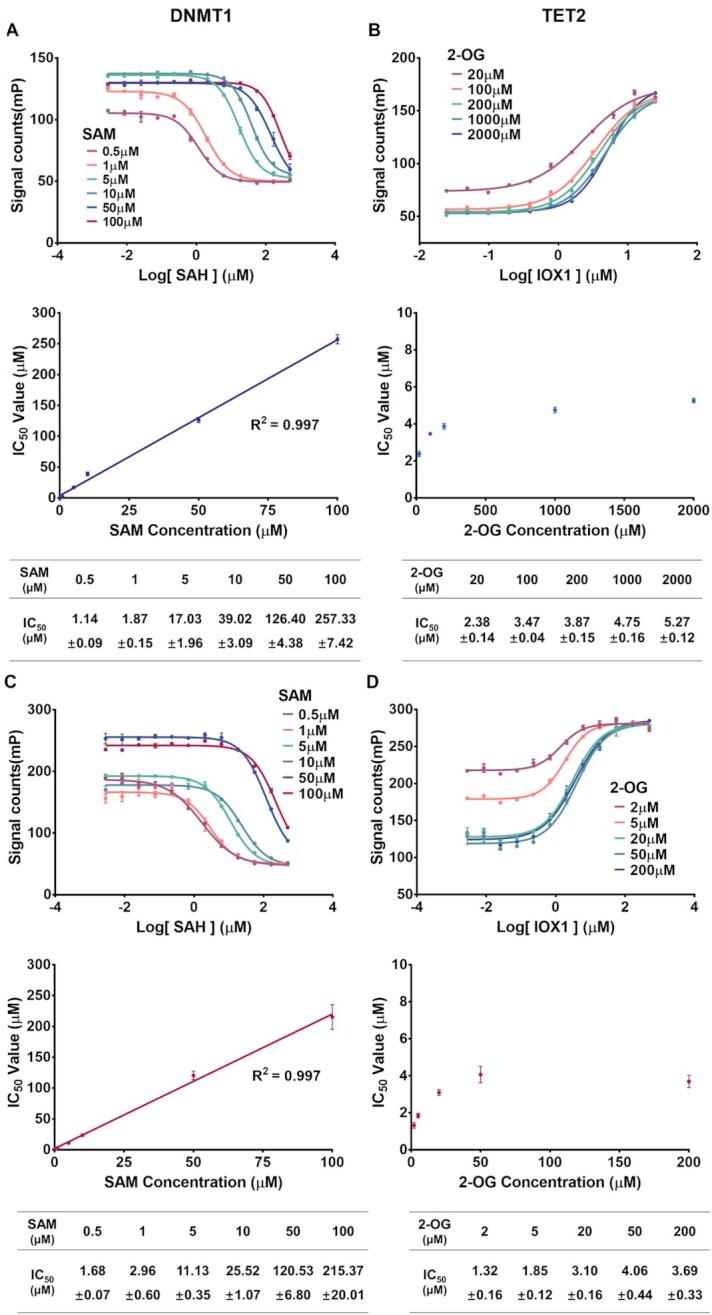
Inhibition curves of SAH (a natural competitive inhibitor of methyltransferases) to (**A**) DNMT1, (C)METTL3/14, and IOX1 (spectrum competitive inhibitor of 2-OG dependent dioxygenases) to (**B**) TET2 and (D)ALKBH5. Reactions were performed at different concentrations of SAM or 2-OG, in triplicate. Error bars are displayed as mean ± SD (*n* = 3).

Targeting the substrate-binding pocket is an effective strategy for drug discovery. Cofactor-competitive inhibitors of epigenetic enzymes e.g.A485 (21), WM-8014 (27), EPZ5676 (20), EPZ-6438 (19), GSK2807 (25) and GSK-J1 (28) have been developed and are potent and selective in both *in vitro* and *in vivo* studies. It is necessary to evaluate the binding mode and potential binding sites of candidate compounds with high throughput screening (19–21). An FP binding assay of the enzyme can be used to analyze oligonucleotide competition, while our assay should perform cofactor competition since this detection is independent of the concentration of cofactors. SAH (a natural competitive inhibitor of methyltransferase) and IOX1 (75) (a spectrum competitive inhibitor of 2OG-dependent dioxygenase) were used to perform a competition assay of DNMT1, METTL3/14 and TET2, ALKBH5. The IC_50_ value of SAH against DNMT1 and METTL3/14 increased linearly along with increasing concentrations of SAM with *R*^2^ greater than 0.99, further confirming the accuracy of the assay (Figure 4A, C). The *K*_i_ of SAH (3.63 ± 3.13 μM for DNMT1, 2.06 ± 2.80 μM for METTL3/14, respectively) could be calculated as described (64), which is close to previous studies (71,76).

The IC_50_ value of IOX1 against ALKBH5 is also increasing and is close to the previous study as measured by radioisotopes at 5, 10 and 20 μM of 2-OG (71) (Figure 4D). However, a higher concentration of 2-OG did not further increase the IC_50_ in our study. This could be because the 2-OG competitive inhibitor IOX1 forms a stable interaction of coordination bonds of metal ions (75) or the depletion of Fe^2+^. The same appearance was observed in the TET2 demethylation reaction (Figure 4C). Conclusively, our assay is reliable for evaluating the enzymatic inhibitory activity of both DNA and RNA methylation/demethylation enzymes.

### The assay is highly feasible for high throughput screening

**Figure 5.**
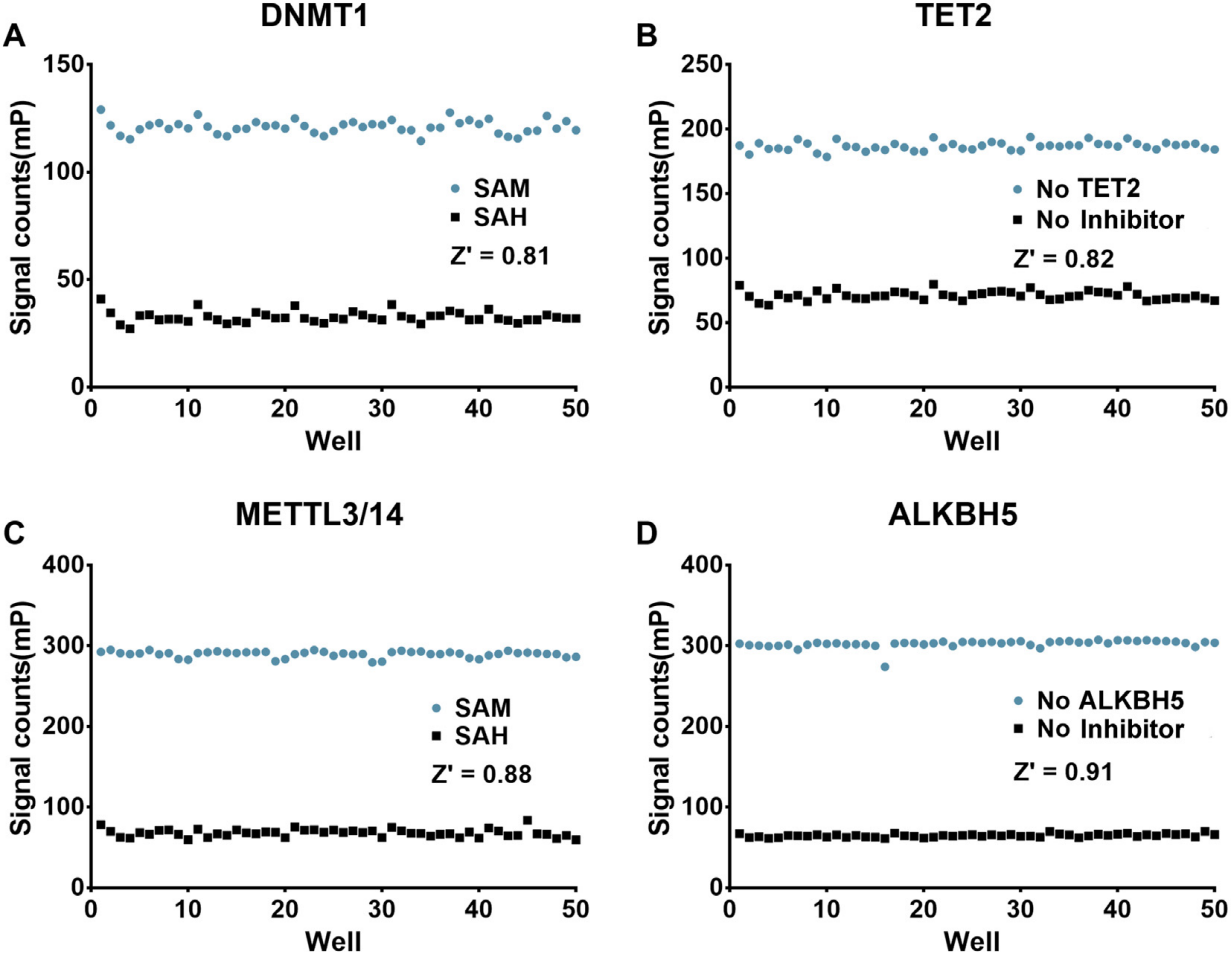
Z’ factor determination of the assay for (**A**) DNMT1, (**B**)TET2, (**C**) METTL3/14 and (**D**) ALKBH5. SAM (SAH) concentration used in the assay was 1 μM (10 μM) for DNMT1 and METTL3/14. The 2-OG concentration was 1 mM and 200 μM for TET2 and ALKBH5, respectively.

A high-throughput assay should be cheap and simple. Our HTMR assay is quick and simple to operate. The FP signals can be measured in a single mix-and-read step without a long incubation time, as follows: First, incubate the compounds with the enzyme and then add the substrate to initiate the reaction. Second, add methyl-binding proteins into the wells and incubate for 10–15 min after the reaction is finished. Third, measure the signal with a Multimode Plate Reader using a fluorescence polarization standard procedure. To evaluate the performance of our assay, we determined the Z’ factor (63) to be 0.82, 0.80, 0.88, and 0.91 for DNMT1 (at 1 μM SAM), TET2 (at 1 mM 2-OG), METTL3/14 (at 2.5 μM SAM) and ALKBH5 (at 200 μM 2-OG), respectively. These values are robust and far greater than the required value of 0.5 (Figure 5). Our assay is highly sensitive and can be performed at low concentrations of enzymes and oligonucleotide substrates (below 50 nM). The concentration of the cofactors must also be low enough to screen weak cofactor-competitive inhibitors. All assay materials are easy for a biochemistry laboratory to obtain. The tri-MBD1 and YTHDF1 proteins can be effectively expressed in *E. coli* with a very high yield (over 30mg per liter medium). The relatively high-cost reagent is a fluorescent-labeled oligonucleotide (Supplementary Table S1), which is easy to purchase from a biotech company. In summary, our assay is simple, robust, and cost-effective, all of which are necessary for large-scale screening.

### Identification of DNMT1 and ALKBH5 inhibitors by high throughput screening based on HTMR assay

**Figure 6.**
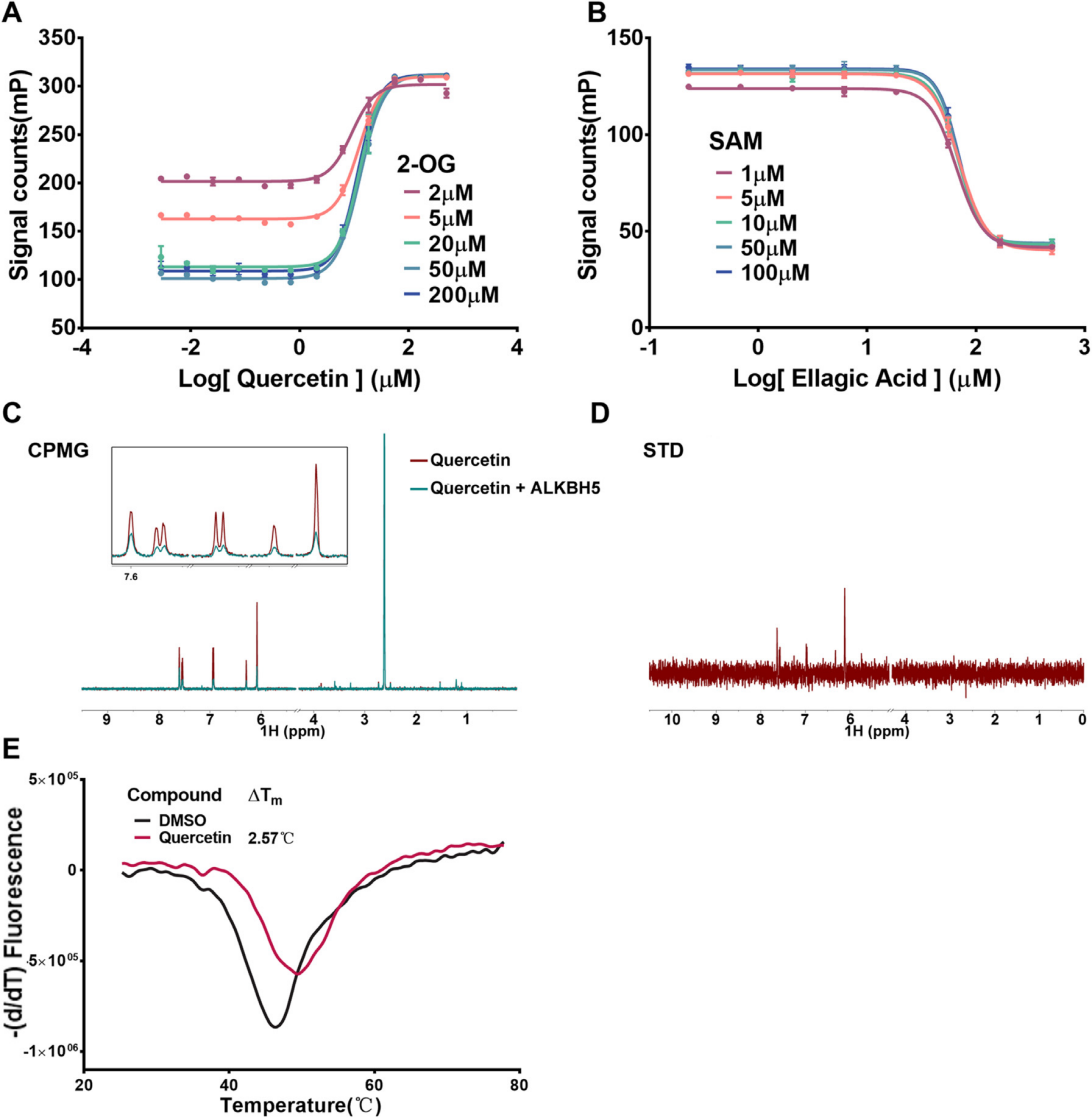
Primary evaluation of candidate compounds in the screening. Inhibition curve of (**A**) quercetin (ALKBH5) and (**B**) ellagic acid (DNMT1). Reactions were performed at different concentrations of co-factors, in triplicate. Error bars are displayed as mean ± SD (*n* = 3). (**C**) CPMG spectra for 200 μM compound quercetin without (red) or with (cyan) the presence of 5 μM ALKBH5. (**D**) STD spectra for 200 μM quercetin in the presence of 5 μM ALKBH5. (**E**) Thermal shift assay of ALKBH5. Derivative melting curves of 5 μM ALKBH5 were plotted in the presence of compounds or DMSO. Quercetin displayed stabilization of ALKBH5 at a molar ratio of 1:20.

Finally, we performed high-throughput screening against DNMT1 and ALKBH5 for an in-house library of 2000 compounds. False-positive compounds that could interfere with fluorescence or disrupt the binding of tri-MBD1 and YTHDF1 were excluded by a counter screen (specific details of high-throughput screening and counter screen are displayed in Supplementary Figure S7 and the Materials and Methods section). Half of the substrate oligonucleotide was replaced with the product (chemical synthetic) in the assay (the enzyme was removed), in which case the false-positive compounds would still influence the FP signal value while the true hits would not. In this study, we identified two known natural products as promising inhibitors: ellagic acid for DNMT1 and quercetin for ALKBH5 (Figure 6A, B). We performed a cofactor-competitive assay to evaluate the biological activity and mechanism of action of hit compounds. The IC_50_ of ellagic acid is barely affected by increasing concentrations of SAM, indicating a SAM-noncompetitive mechanism with IC_50_ value of approximately 67 μM. The DNMT1 FP binding assay revealed that ellagic acid competed with the binding of DNA (Supplementary Figures S4A, S5A) and the IC_50_ value of quercetin in the ALKBH5 reaction did not significantly change at different concentrations of 2-OG (Supplementary Figure S4B). The compound did not affect the RNA binding of ALKBH5 in the ALKBH5 FP binding assay, indicating an allosteric regulation mechanism (Supplementary Figure S5B). Quercetin also demonstrated high activity during the TET2 demethylation reaction (Supplementary Figure S4C, D). A protein thermal shift assay demonstrated that quercetin could bind and stabilize ALKBH5 with a *T*_m_ shift of 2.57°C (Figure 6E). Furthermore, we performed an NMR study of Carr-Purcell-Meiboom-Gill (CPMG) and the saturation transfer difference (STD), which revealed that quercetin is strongly bound to the ALKBH5 catalytic domain directly (Figure 6C, D). In summary, our method is feasible to screen large-scale collections of screen compounds and evaluate the biological activity of hit compounds.

## DISCUSSION

Small molecules that regulate DNA/RNA methylation have a wide range of possible applications. DNMTi—Vidaza and Decitabine are approved drugs that can robustly reverse global hypermethylation of DNA and have been used in various biological and clinical studies (36,41,44). The restoration and activation of TETs are regulated by small molecular agonists that could provide promising therapy in cancer treatments via DNA demethylation (32,77). Ascorbic acid, which strongly improves demethylation activity in a Fe^2+^-independent manner, can directly bind TETs and help block the progression of leukemia (32,47). The m^6^A regulator proteins are another promising target drug and have recently attracted the attention of researchers in various biological fields (30,31,35).

While there is significant potential for drug development for DNA/RNA regulatory enzymes, little progress has been made and only a few potent small molecules have been reported. This is partly due to a lack of effective high-throughput screening methods. Radioisotopes are the gold standard assay for methyltransferases, but require strict experimental conditions and generates radioactive waste. HPLC analysis is the assay typically used for a demethylation reaction but is time-consuming, inefficient, and expensive. Other methods have different shortcomings, including those which are insensitive, non-homogenous, and incapable of being used in competition studies. Most are unable to perform large-scale screenings.

In this study, we developed a homogeneous HTMR assay to perform high-throughput screening for DNA/RNA methyltransferases and demethylases by coupling the FP binding assay of methyl-binding proteins. The feasibility of this assay was first demonstrated by theoretical calculations, in which *K*_a_/*K*_b_ and mP_max_–mP_min_ were used to assess assay performance. We then demonstrated the variable selectivity of the methyl-binding proteins towards methyl-oligonucleotides under different salt concentrations. In these cases, high ionic strength prevents the enzyme from binding to the oligonucleotides. It is therefore necessary to optimize the proper composition of the assay buffer to obtain appropriate signals for compound screening. Accordingly, we conducted a series of enzyme assays on DNMT1, TET2, METTL3/14 and ALKBH5 to confirm that the assay is stable enough to perform high-throughput screening with robust *Z*′ factors greater than 0.8. We evaluated the biological activity of the known inhibitors-SAH and IOX1 (75), which were very close to the results previously reported via radioisotope. The HTMR assay can easily be used to conduct a substrate competition study of lead compounds, which is important for evaluating the action mechanism. To demonstrate the practical applicability of this method, we screened a collection of 2000 in-house compounds and successfully identified two natural products as candidate compounds for DNMT1 and ALKBH5: ellagic acid and quercetin, respectively.

Unlike the typical radioisotope, HPLC, and ELISA methods, our assay is homogeneous, simple to perform, and capable of high-throughput screening. It also uses fluorescence polarization and has low substrate and enzyme requirements, all of which make the assay highly sensitive and cheap. High throughput screening methods usually produce false positives, including our assay. These compounds could influence the fluorescence or binding of methyl-binding proteins, which must be excluded by counter screening. This study provides a powerful assay method and allows for the mass screening of large and diverse compound libraries to meet drug discovery requirements of DNA/RNA methyl modification regulatory enzymes. This assay can help identify more potent small-molecule regulators of DNA/RNA methylation-related enzymes and establish drug candidates for therapeutic application in diseases caused by the dysregulation of DNA/RNA methylation-related enzymes.

## DATA AVAILABILITY

All the raw data including NMR, HPLC and the original files generated by EnVision have been deposited in the DRYAD database at https://doi.org/10.5061/dryad.f7m0cfxw9. All other data supporting the findings of this study are available upon request.

## Supplementary Material

gkab989_Supplemental_File

## Notes

Present address: Shijie Chen, The Center for Chemical Biology, Drug Discovery and Design Center, Shanghai Institute of Materia Medica, CAS, Zuchongzhi Road 555, Shanghai 201203, China.
