# Supplementary material for: High-Throughput-Methyl-Reading (HTMR) assay: a solution based on nucleotide methyl-binding proteins enables large-scale screening for DNA/RNA methyltransferases and demethylases
Source: Nucleic Acids Res. 2021 Oct 29;50(2):e9. doi: 10.1093/nar/gkab989 (PMC8789064; doi:10.1093/nar/gkab989)
Supplement: gkab989_Supplemental_File [file gkab989_supplemental_file.pdf]

Supplemental Information

High-Throughput-Methyl-Reading (HTMR) Assay: a solution based on Nucleotide Methyl-Binding Proteins enables large-scale screening for DNA/RNA Methyltransferases and Demethylases

Senhao Xiao[1,2,3], Siqi Guo[1], Jie Han[1], Yanli Sun[1,6], Mingchen Wang[1,2,3], Yantao Chen[1], Xueyu Fang[1,2,3], Feng Yang[1,5], Yajuan Mu[7], Liang Zhang[7], Yiluan Ding[3,4], Naixia Zhang[3,4], Hualiang Jiang[1,2,3], Kaixian Chen[1,2,3], Kehao Zhao[6], Cheng Luo[1,3,4,8] and Shijie Chen[1,3,4]*

[1]The Center for Chemical Biology, Drug Discovery and Design Center, State Key Laboratory of Drug Research, Shanghai Institute of Materia Medica, Chinese Academy of Sciences, Shanghai, 201203, China

[2]School of Life Science and Technology, Shanghai Tech University, Shanghai 201210, China

[3]University of Chinese Academy of Sciences, No.19A Yuquan Road, Beijing 100049, China

[4]CAS Key Laboratory of Receptor Research, Shanghai Institute of Materia Medica, Chinese Academy of Sciences, Shanghai 201203, China.

[5]School of Pharmacy, Fudan University, Shanghai, 201203, China

[6]School of Pharmacy, Key Laboratory of Molecular Pharmacology and Drug Evaluation (Yantai University), Ministry of Education; Collaborative Innovation Center of Advanced Drug Delivery System and Biotech Drugs in Universities of Shandong, Yantai University, Yantai 264005, China

[7]Department of Pharmacology and Chemical Biology, State Key Laboratory of Oncogenes and Related Genes, Shanghai Jiao Tong University School of Medicine, Shanghai, 200025, China

[8]School of Pharmaceutical Science and Technology, Hangzhou Institute for Advanced Study, UCAS, Hangzhou 310024, China

* To whom correspondence should be addressed. Tel:86-021-50806600;

Email:shijiechen@simm.ac.cn;

Present Address: Shijie Chen, Drug Discovery and Design Center, Shanghai Institute of Materia Medica, CAS, Zuchongzhi Road 555, Shanghai, 201203, China.

.

Table S1. The oligonucleotides used in this study.

12bp-CpG containing dsDNA	Sequence	
unmethylated dsDNA- [C/C]	top strand: 5′-FAM-TACGACCAGGAT-3′<br>bottom strand: 5′-ATCCTGGTCGTA-3′	
hemi-methylated dsDNA- [M/C]	top strand: 5′-FAM-TA5mCGACCAGGAT-3′<br>bottom strand: 5′-ATCCTGGTCGTA-3′	substrate for DNMT1
fully-methylated dsDNA-[M/M]	top strand: 5′-FAM-TA5mCGACCAGGAT-3′<br>bottom strand: 5′-ATCCTGGT5mCGTA-3′	product for DNMT1<br>substrate for TET2
fully-hydroxymethylated dsDNA-[H/H]	top strand: 5′-FAM-TA5hmCGACCAGGAT-3′<br>bottom strand: 5′-ATCCTGGT5hmCGTA-3′	main product for TET2
fully-carboxylated dsDNA-[caC/caC]	top strand: 5′-FAM-TA5caCGACCAGGAT-3′<br>bottom strand: 5′-ATCCTGGT5caCGTA-3′	final product for TET2
16nt GGACU motif containing RNA	Sequence	
unmodified RNA	5′-FAM-GAACCGGACUGUCUUA-3′,	substrate for METTL3/14<br>product for ALKBH5
m6A-modified RNA	5′-FAM-GAACCGGm6ACUGUCUUA-3′,	substrate for ALKBH5<br>product for METTL3/14

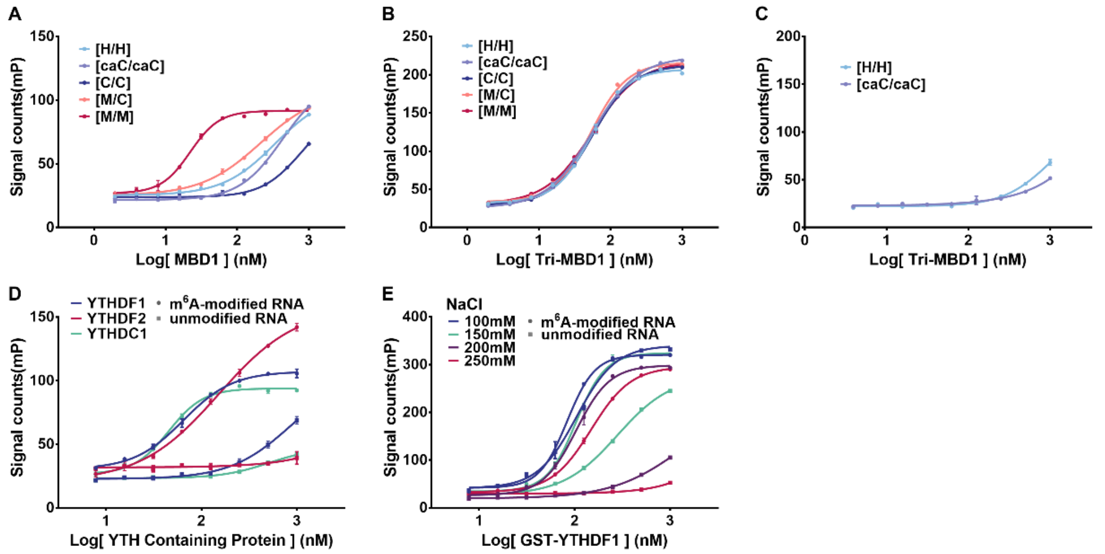

Figure. S1. Titration of the fluorescence-labeled oligonucleotides at different modification statuses with methyl-binding proteins. (A) untagged MBD1 with [C/C] (unmethylated DNA), [H/H] (fully-hydroxymethylated DNA), [caC/caC] (fully-carboxylated DNA), [M/C] (hemi-methylated DNA), and [M/M] (fully-methylated DNA) in 150 mM NaCl, (B) tri-MBD1 with [C/C], [H/H], [caC/caC], [M/C], and [M/M] in 150 mM NaCl. (C) tri-MBD1 with [H/H] and [caC/caC] in 300 mM NaCl. M, H, caC, C, represent methylated, hydroxymethylated, carboxylated, and unmodified cytosine in dsDNA. (D) YTH domain containing proteins-YTHDF1, YTHDF2, YTHDC1 with m^6A modified RNA and unmodified RNA in 150 mM NaCl. (E) GST-YTHDF1 with m^6A modified RNA and unmodified RNA in different concentrations of NaCl. All samples were performed in triplicate. The error bars were showed as mean ± SD (n = 3).

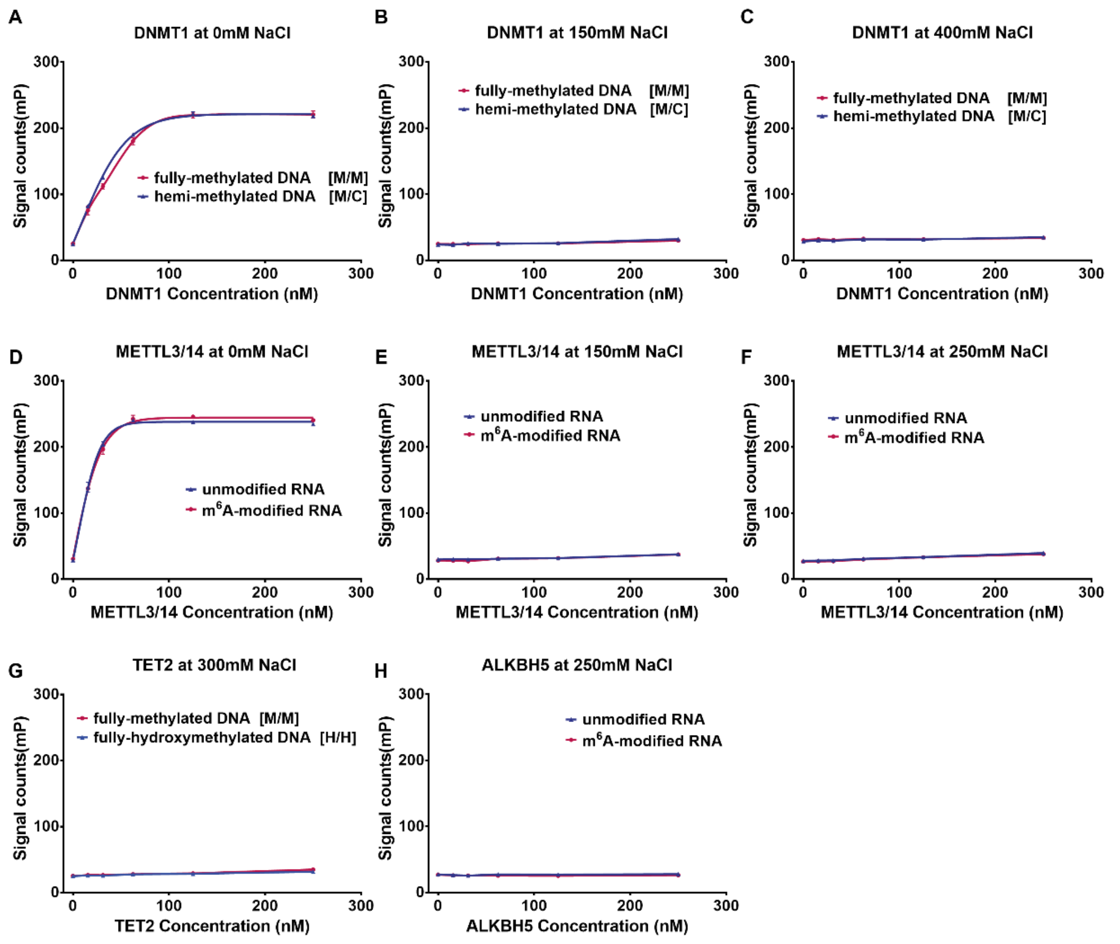

Figure. S2. FP binding curves of the enzymes. DNMT1 in a buffer containing (A) 0 mM, (B) 150 mM, (C) 400 mM NaCl. METTL3/14 in a buffer containing (D) 0 mM, (E) 150 mM, (F) 250 mM NaCl. (G) TET2 in a buffer containing 300 mM NaCl. (H) ALKBH5 in a buffer containing 250 mM NaCl. All samples were performed in triplicate. The error bars were showed as mean ± SD (n = 3).

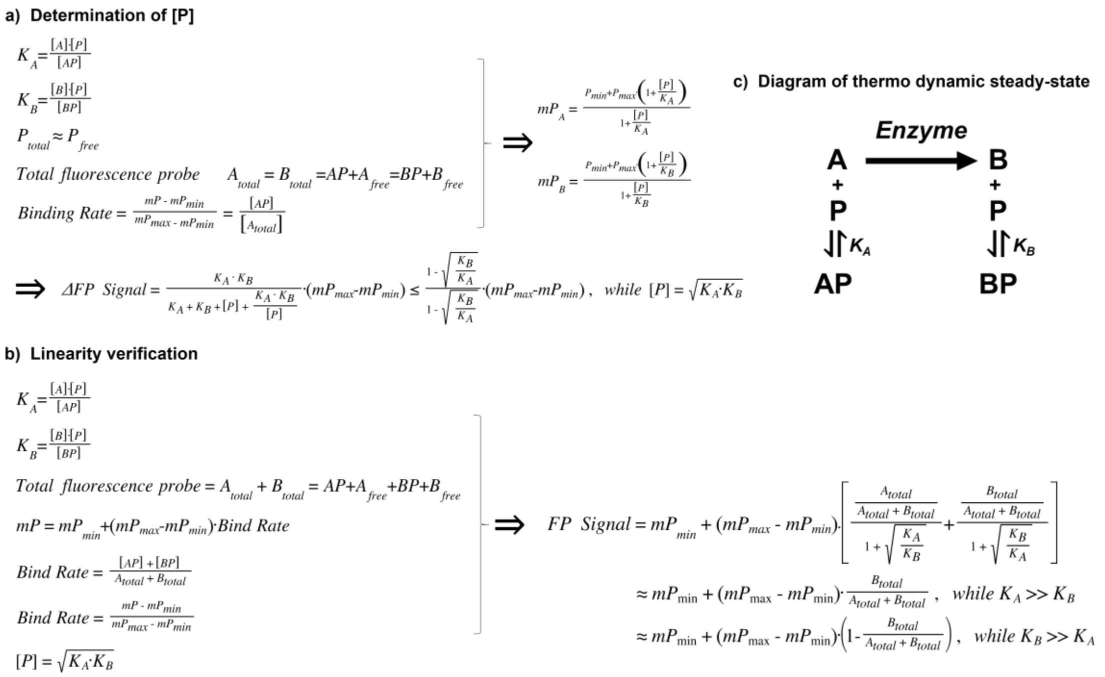

Figure. S3. Equation derivation according to the binding model of methyl-binding protein while reaction proceeds. The calculation ignores the impact of enzyme bind to the fluorescence-labeled oligonucleotides. In the equations, A, B, P represent substrate, product, and methyl-binding proteins, respectively. AP and BP represent the association form. K_A and K_B represent the dissociation constant of substrate and product, respectively. mP_{max} and mP_{min} represent the maximum and minimum FP signal in the FP binding assay. [P] is the concentration of methyl-binding protein in free-state. P_{total}, A_{total}, and B_{total} are the sum of free from and association form of P, A, and B, respectively. $\frac{B_{total}}{A_{total}+B_{total}}$ represents the percentage of substrate conversion. *ΔFP signal* represent the signal window (dynamic range) of the assay. *FP signal* represent fluorescence polarization signal value of the system.

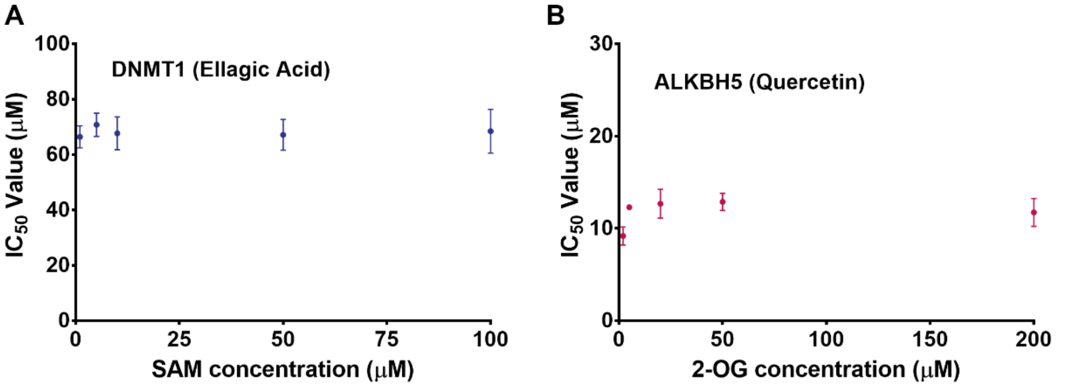

SAM (μM)	1	5	10	50	100
IC_{50} (μM)	66.44 ±3.98	70.80 ±4.21	67.71 ±5.93	67.15 ±5.60	68.50 ±7.94

2-OG (μM)	2	5	20	50	200
IC_{50} (μM)	9.19 ±0.99	12.30 ±0.21	12.69 ±1.57	12.89 ±0.94	11.75 ±1.51

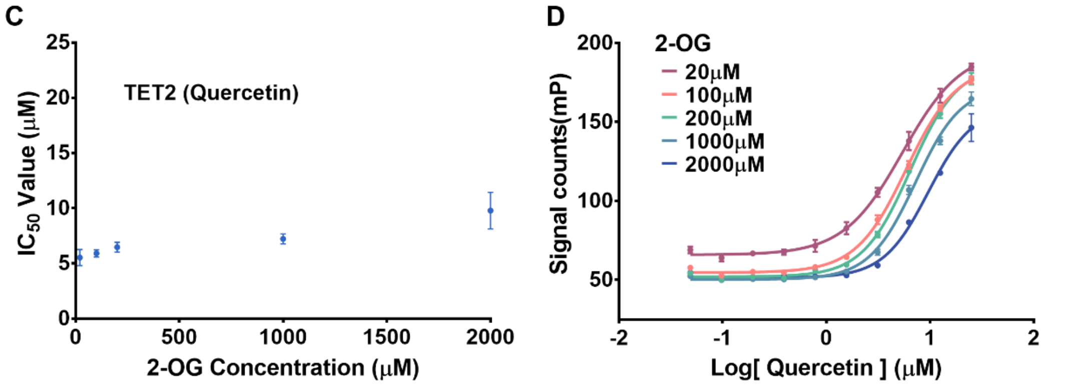

2-OG (μM)	20	100	200	1000	2000
IC_{50} (μM)	5.53 ±0.74	5.91 ±0.33	6.47 ±0.45	7.22 ±0.44	9.79 ±1.66

Figure. S4. The IC_{50} of (A) ellagic acid in DNMT1 assay, (B) quercetin in ALKBH5 assay, (C) quercetin in TET2 assay and (D) quercetin inhibition curves of TET2. The reactions were performed in triplicate. The error bars were showed as mean ± SD (n = 3).

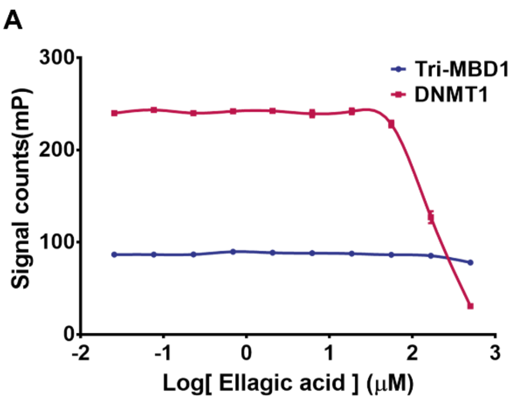

Figure. S5. Counter screen and enzyme binding assay of hit compounds. (A) Ellagic acid didn't interference the assay (blue lines), and disturbed the DNA-DNMT1 interaction in the FP binding assay of DNMT1 (red lines). (B) Quercetin didn't interference the assay (blue lines). And the compound didn't affect the RNA binding of ALKBH5 (red lines). All samples were performed in triplicate. The error bars were showed as mean ± SD (n = 3).

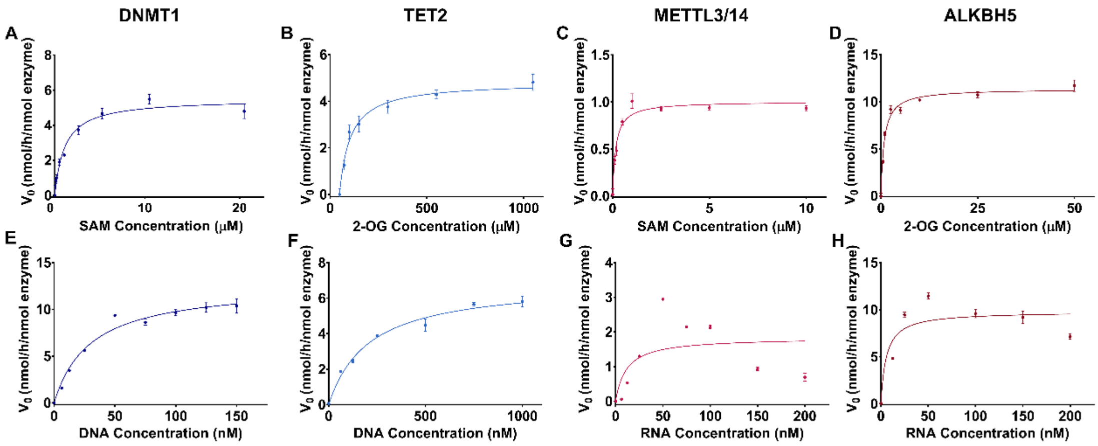

I

Enzyme	Substrate	K_m (μM)	k_{cat} (h^{-1})
DNMT1	SAM	1.06±0.14	12.7±0.47
	DNA	0.030±0.0037	
TET2	2-OG	55.52±7.62	6.92±0.24
	DNA	0.207±0.022	
METTL3/14	SAM	0.162±0.024	1.01±0.03
	RNA	—	
ALKBH5	2-OG	0.843±0.079	11.36±0.21
	RNA	—	

Figure. S6. The kinetic parameters determination of DNMT1 (A) for SAM, (E) for DNA, TET2 (B) for 2-OG, (F) for DNA, METTL3/14 (C) for SAM, (G) for RNA, and ALKBH5 (D) for 2-OG, (H) for RNA. (I) The table is the summary of the measurements. All samples were performed in triplicate. The error bars were showed as mean ± SD (n = 3).

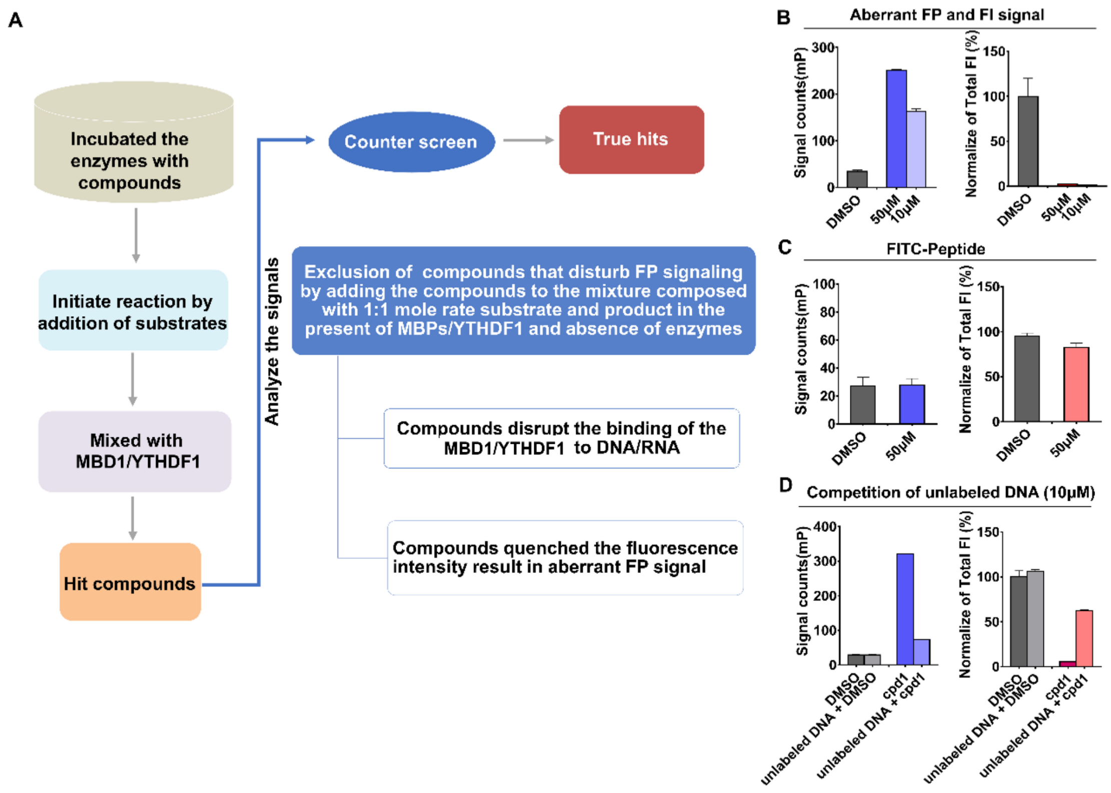

Figure. S7. (A) Workflow of screening and counter screening. (B) Cpd1 is a typical example compound we found that interfere the assay (DNA binder). The cpd1 interfere the assay and resulted in aberrant FP signal and fluorescence quenching (FI signal) in the presence of only compounds and DNA without enzymes. (C) The cpd1 have no significant interference to both FP and FI value of an N terminal FITC-labeled FSSNRQKILERTETLNQEWKQR peptide. (D) The aberrant FP signal and fluorescence quenching caused by cpd1 could be reversed by 10 μM unlabeled DNA.
